# Coffee consumption and risk of esophageal cancer incidence: A meta-analysis of epidemiologic studies

**DOI:** 10.1097/MD.0000000000010514

**Published:** 2018-04-27

**Authors:** Juan Zhang, Bin Zhou, Chuanzheng Hao

**Affiliations:** a Department of Rehabilitation; b Department of Hepatobiliary Surgery, Research Institute of Hepatobiliary Surgery of Nantong University, Affiliated Hospital Of Nantong University, Nantong, Jiangsu Province, China.

**Keywords:** coffee, esophageal cancer, meta-analysis

## Abstract

**Background:**

In epidemiologic studies, association between coffee consumption and esophageal cancer risk is inconsistent.

**Objective:**

The aim of tjis study was to evaluate the effect of coffee on esophageal cancer by combining several similar studies.

**Methods:**

We conducted a meta-analysis for association of coffee intake and esophageal cancer incidence. Eleven studies, including 457,010 participants and 2628 incident cases, were identified. A relative risk (RR, for cohort study) or odds ratio (OR, for case–control study) of heavy coffee drinkers was calculated, compared with light coffee drinkers or non-drinkers. The analysis was also stratified by cancer types (esophageal squamous cell carcinoma and esophageal adenocarcinoma), sex, and geographic region.

**Results:**

The summarized OR of having esophageal cancer in heavy coffee drinkers was 0.93 (95% confidence interval [CI]: 0.73–1.12), compared with light coffee drinkers. When stratified by sex, pathologic type of esophageal cancer, and type of epidemiologic study, we did not find any association of coffee consumption and esophageal cancer incidence. However, an inverse association between coffee consumption and incidence of esophageal cancer was found in East Asia participants with OR of 0.64 (95% CI: 0.44–0.83), but not in Euro-America participants (OR = 1.05; 95% CI: 0.81–1.29).

**Conclusion:**

There is a protective role of coffee consumption against esophageal cancer in East Asians, but not in Euro-Americans.

## 1 Introduction

Esophageal cancer is a major concern in the world, ranking the sixth most common cause of cancer mortality.^[1]^ Lifestyles such as smoking, drinking, and dietary habits have been suggested to be associated with the carcinogenesis of esophageal cancer.^[2,3]^ Coffee is considered to decrease the incidence of cancer through the effect of its anticarcinogenic constituents such as polyphenols (chlorogenic acid, caffeic acids, among others) and diterpenes (cafestol and kahweol).^[4–6]^ A number of epidemiologic studies, including several cohort studies and many case-–control studies, have evaluated the relationship of coffee consumption and esophageal cancer risk in human beings, but their findings are inconsistent. As many researchers in the world, we are interested in relationship of coffee consumption and the risk of esophageal cancer incidence because coffee is one of the most widely consumed beverages in the world and incidence and mortality of esophageal cancer are high worldwide.^[7]^ Therefore, we conducted this meta-analysis to examine the association in epidemiologic studies.

## 2 Material and methods

The electronic databases, PubMed (1966 to January 2017), Embase (1980 to January 2017), the Science Citation Index (1945 to January 2017), and the Chinese Biomedical Database (1981 to January 2017) were searched for studies of coffee consumption in relation to esophageal cancer risk, which were published in both English or Chinese. We used terms of “coffee,” or “caffeine” combined with medical-subject-heading terms “esophageal neoplasms,” “oesophageal neoplasms,” or “esophagus,” or keywords “esophageal cancer” or “oesophageal cancer” in the paper search. All analyses were based on previous published studies; thus, no ethical approval and patient consent are required.

## 3 Inclusion criteria

1. Type of study: observational studies: including case-control study and cohort study.
2. Exposure factor: coffee consumption.
3. Statistical indicators: Relative risk (RR, for cohort study) or odds ratio (OR, for case–control study) of heavy coffee drinkers, compared with light coffee drinkers or nondrinkers, was presented.

## 4 Data extraction

We extracted the data for the first author's name, publication year, the country of origin, duration of follow-up, sex, the number of participants (cases and cohort size), measurements of coffee consumption and OR estimates, and their corresponding 95% CIs. When a study provided ORs for both esophageal cancer and invasive esophageal cancer, we used the former because of having more cases. Two reviewers (JZ and BZ) independently inspected the data for eligibility and quality. Disagreements were resolved by the third reviewer (CH).

## 5 Statistical analysis

The Stata version 12.0 software (Stata Corporation, College Station, TX) was used to analyze the OR or RR. Analysis was stratified by cancer types, sex, and geographic region to exclude effect of possible confounders. In the meta-analysis, heterogeneity across the studies was examined using the Q statistic. Statistical significance was set at 0.10 for heterogeneity.^[8,9]^ If *P* value of heterogeneity was <.10, the random model was used instead of the fixed-effect model for further analysis. Funnel plots with the Begg test^[10]^ and Egger^[11]^ test were visually evaluated to detect a possible publication bias with α = 0.1011. We pooled the ORs for highest versus lowest categories of coffee consumption from each study. We combined data of 11 studies and estimated a summary OR of coffee consumption and esophageal cancer risk. Subgroup analyses were performed by sex, geographic region, pathologic type of esophageal cancer, and type of epidemiologic studies.

## 6 Results

### 6.1 Search results

**Figure 1:**
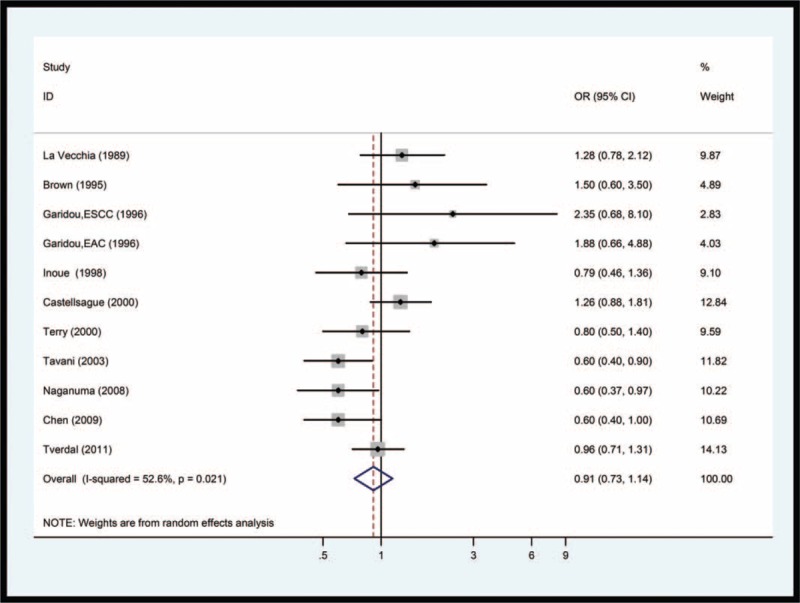
Forest plot of studies of the risk of esophageal cancer for the highest versus lowest coffee consumption. The size of the data markers (squares) corresponds to the weight of the study in the meta-analysis. The combined odds ratio is calculated using the random-effects method.

In our initial retrieve, 3599 articles were identified, but 3572 articles were excluded for lack of clinical trial (n = 2987), duplication (n = 285), and unrelated statistical indicators (n = 300). After a detailed assessment of full texts, 9 were excluded because of insufficient data and 7 articles were excluded because of being nonobservational studies. Finally, 11 trials were included in this meta-analysis (Fig. 1).

### 6.2 Characteristics of included studies

Eleven epidemiologic studies^[12–21]^ including 457,010 participants and 2628 incident cases of esophageal cancer were identified according to the inclusion criteria in the meta-analysis. The characteristics of the included studies are summarized in Table 1. Three studies^[15,19,20]^ were conducted in East Asia (640 cases), whereas another 8^[12–14,16–18,21]^ studies were conducted in Europe and America (1988 cases). Two studies conducted in Japan^[19]^ and Norway^[21]^ were cohort studies and the others were case–control studies. Only 2 studies^[16,21]^ provided sex-specific ORs, but 1 study^[20]^ from Taiwan included male participants only. Therefore, the 3 studies were used for male esophageal cancer (1218 cases) and 2 studies for female esophageal cancer (269 cases), respectively.

**Table 1:**
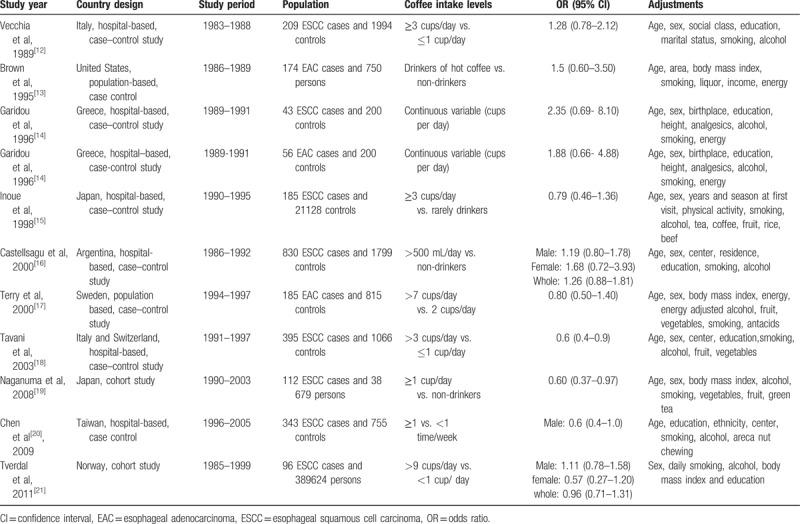
Characteristic of including studies of coffee consumption and incidence risk of esophageal cancer.

### 6.3 Highest versus lowest category of coffee consumption with esophageal cancer risk

A combined OR of highest coffee consumption was 0.93 (95% CI: 0.73–1.12), compared with lowest coffee consumption (Fig. 1). Risk estimates stratified by sex, geographic region, pathologic type of esophageal cancer, and type of epidemiologic studies are shown in Table 2. There was no association between coffee consumption and the risk of esophageal cancer among males (OR = 0.94; 95% CI: 0.54–1.33) or in females (OR = 0.66; 95% CI: 0.21–1.10), respectively. There was association between coffee intake and incidence of esophageal cancer neither in prospective cohort study (OR = 0.78; 95% CI: 0.43–1.13) nor in case–control study (OR = 0.98; 95% CI: 0.74–1.22). Comparing highest versus lowest coffee consumption levels for esophageal squamous cell carcinoma (ESCC) and esophageal adenocarcinoma (EAC), the pooled ORs were 0.871 (95% CI: 0.67–1.08) and 1.15 (95% CI: 0.88–1.42), respectively. However, an inverse relationship was found in East Asian population (OR = 0.64; 95% CI: 0.44–0.83), but not in Euro-American population (OR = 1.05; 95% CI: 0.81–1.29).

**Table 2:**
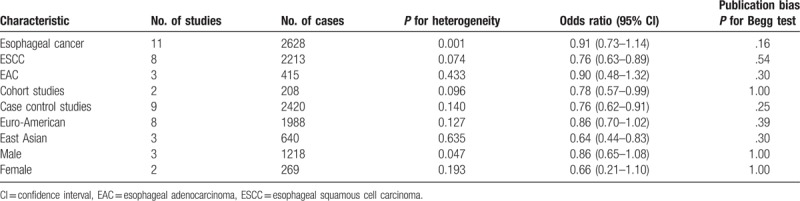
Association of coffee consumption and esophageal cancer risk by sex, geographic region, pathologic type of esophageal cancer, and type of epidemiologic studies.

### 6.4 Publication bias

**Figure 2:**
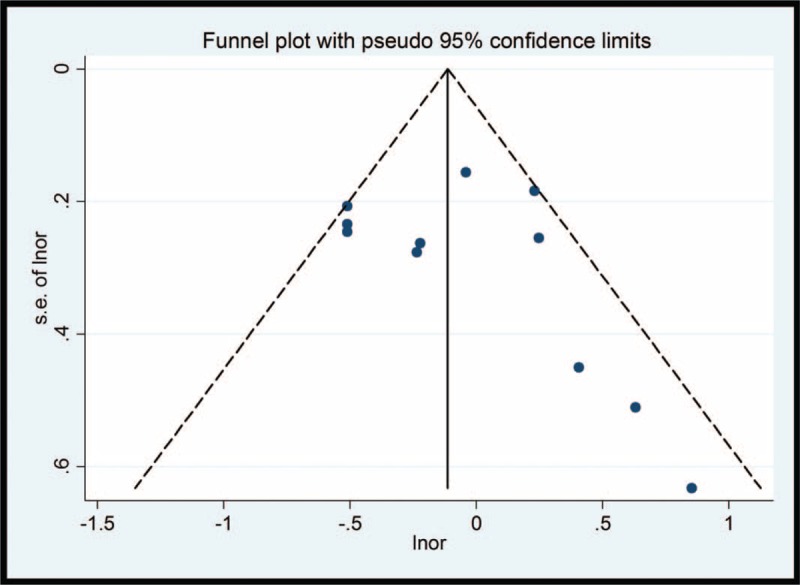
Begg funnel plot of studies on coffee consumption and esophageal cancer risk. The solid line in the center is the natural logarithm of pooled odds ratio, and 2 oblique lines are pseudo 95% confidence limits. SE = standard error.

There was no evidence of publication bias in the overall trials (Fig. 2) and the trials in each subgroup (all *P* >.1 for Begg test and Egger test), with an exception of the ESCC subgroup (*P* = .058 for Egger test).

## 7 Discussion

In general, our meta-analysis did not find association between coffee consumption and risk of esophageal cancer incidence. However, East Asian who drank coffee every day had a lower incidence of esophageal cancer than those who rarely or never drank coffee, which may be caused by different prevalence of esophageal cancer between Euro-American and East Asian countries. The cumulative rate of EAC is higher in Euro-America than in East Asia. The former is 0.01 to 1.19 and the latter is 0 to 0.15.^[22]^ The cumulative rate of ESCC is highest among East Asians, and lowest in white subjects.^[22]^ However, ESCC is the dominant type of esophageal carcinomas in Asian countries, especially in China^[22]^ including Hong Kong^[23]^ and Taiwan,^[24]^ Korea,^[25]^ and Japan.^[26,27]^ The ratio of EAC among esophageal malignancies was as low as 1% to 4% in Korea,^[25]^ Taiwan,^[24]^ and Japan.^[27,28]^ Therefore, overall incidence of esophageal cancer is much higher in East Asia than that in Europe and America. Coffee consumption may play a more protective role of incidence of esophageal cancer in high-risk area, such as East Asia.

A number of experimental and clinical studies have suggested that drinking very hot beverages may be a cause of esophageal cancer. Evidences that tumors were larger sizes of esophageal papillomas rapidly increased when people took hot food with the temperature at 70°C and above and this relationship was confirmed by an experimental study.^[29]^ In our meta-analysis, only 1 study ^[16]^ investigated the association between temperature of coffee drinking and risk of esophageal cancer incidence and concluded that higher temperature of coffee did not increase incidence of esophageal cancer. Owing to little information on relation of temperature of coffee drinking and esophageal cancer, it was difficult to exclude interference from temperature of coffee drinking.

Six studies^[12,14,15,17,18,21]^ included in the present study have investigated the effects of coffee drinking and dose–response relationships and 5 of them did not find dose–response relationships.^[12,14,15,17,21]^ In the study conducted by Tavani et al,^[18]^ more coffee consumption daily decreased the risk of getting esophageal cancer (*P* for trend = .007). We did not do analysis stratified by amount of coffee consumption because of the discrepancy of categorization of coffee consumption in those studies.

Smoking is associated with esophageal cancer and stop smoking may reduce the risk of esophageal cancer. Also there is a strong association between alcohol consumption and esophageal squamous cell carcinoma.^[30]^ Four studies^[12,14,18,20]^ included in the meta-analysis have observed the association between cigarette smoking and alcohol drinking and risk of incidence of esophageal cancer. In all 4 studies, cigarette smoking and alcohol drinking were found to be substantially associated with an increased risk of esophageal cancer incidence. All studies included in the meta-analysis provided data adjusted for potential interaction factors among age, smoking, and alcohol, which demonstrated a more independent association between coffee consumption and esophageal cancer incidence.

To minimize the bias, our study referred to many aspects, such as race, sex, age, coffee consumption, and so on. However, there are several disadvantages that should be considered in our meta-analysis. First, the epidemiologic studies did not provide information on the characteristics of the coffee, such as cup size, type of coffee power, and brewing methods, which are responsible for the different concentrations of caffeine and other chemicals in the beverage.^[31]^ Second, almost all original studies have no data on the temperature of the coffee when drinking, which may be a cause of inconsistent data among studies. Finally, although incidence of esophageal cancer is quite high in Africa, we did not find studies conducted among African populations.

In summary, our study suggests that coffee consumption has a protective effect on esophageal cancer risk in East Asian, but not in Euro-Americans. Additional prospective cohort studies should be granted among different populations with carefully control of possible confounders, especially characteristics and temperature of the coffee when drinking.

## Author contributions

**Conceptualization:** Bin Zhou.

**Data curation:** Bin Zhou, Juan Zhang.

**Formal analysis:** Bin Zhou, Juan Zhang.

**Investigation:** Juan Zhang.

**Methodology:** Bin Zhou, Juan Zhang.

**Project administration:** Bin Zhou, Juan Zhang.

**Resources:** Juan Zhang.

**Software:** Bin Zhou, Juan Zhang.

**Supervision:** Chuangzheng Hao.

**Writing – original draft:** Bin Zhou, Juan Zhang.

**Writing – review & editing:** Bin Zhou, Juan Zhang, Chuangzheng Hao.
